# Translating Ayurvedic concepts to modern drug structures: A novel paradigm^☆^

**DOI:** 10.1016/j.jaim.2025.101203

**Published:** 2025-08-04

**Authors:** Antonio Morandi, Maria Cristina Minniti, AN Narayanan Nambi

**Affiliations:** a Ayurvedic Point, Corso Sempione 63, 20149, Milan, Italy; b Ashtamgam Ayurveda Chikitsalayam & Vidyapeedham, 4/495A, Vavanoor, Koottanad, Palakkad, 679 533, India

## Abstract

Modern pharmacology faces challenges in fully explaining inter-individual variability in drug efficacy and side effects. This article introduces a groundbreaking approach that applies Ayurvedic principles to interpret modern drug structures and actions, offering a more comprehensive framework for understanding drug behavior. Through the Collaborative Medicine and Science (Co.M.S.) framework, we demonstrate how Ayurvedic epistemology provides a holistic reading of modern pharmaceuticals using fundamental principles of *Panchamahabhuta*, *Tridosha*, and *Guna*. This Ayurvedic interpretation reveals how drug effects (*Karma*) emerge from the interaction between a substance's inherent qualities (*Guna* profile) and an individual's constitutional context (*Prakriti*/*Vikriti)*, explaining inter-individual variability in drug responses. Illustrative examples using antibiotics and antihypertensives demonstrate how this approach can optimize prescription practices, reduce side effects, and lower healthcare costs. While empirical validation will strengthen this approach, it already offers valuable practical insights that complement—not replace—conventional pharmacology, potentially enhancing personalized medicine and patient outcomes.

## 1 Introduction

Advancements in molecular biology have revolutionized pharmacology, yet significant inter-individual variability in drug response and susceptibility to adverse effects remains a challenge [1,2]. Personalized medicine seeks to address this, often focusing on genomics, but holistic factors related to an individual's systemic functioning are also crucial [3,4]. Traditional medical systems, such as Ayurveda, possess sophisticated frameworks for understanding health, disease, and individual constitution developed over millennia [[5], [6], [7]].

Despite numerous attempts to integrate traditional knowledge with modern biomedicine, most approaches have fragmented traditional concepts and analyzed them through reductionist frameworks, stripping away their holistic context [8,9]. To overcome these limitations, we propose using the Ayurvedic epistemological framework as a primary lens through which to understand modern drug actions—a perspective that provides more comprehensive insights than the mechanistic interpretation based on molecular structure alone.

The Collaborative Medicine and Science (Co.M.S.) framework [[10], [11], [12]] offers a methodology for this integration that preserves the epistemological integrity of both systems while enabling practical clinical applications. This approach does not seek to replace modern pharmacological understanding but rather to support and amplify it by adding deeper layers of interpretation that connect molecular structure to systemic effects.

Our central thesis is that Ayurvedic epistemology provides a more inclusive framework for understanding modern drug actions, revealing how drug effects (*karma*) emerge from the interaction between a substance's guna profile and an individual's constitutional context (*prakriti/vikriti*) [[13], [14], [15]]. This understanding offers immediate practical benefits for clinical practice, enabling more personalized and effective drug prescriptions, while also opening avenues for further research and validation.

This article outlines this conceptual framework and provides illustrative examples, demonstrating pathways for both practical application and future research that could transform our understanding of drug action and personalized medicine.

## 2 The Co.M.S. Framework and foundational Ayurvedic concepts

### 2.1 The Co.M.S. Approach to integration

The Collaborative Medicine and Science (Co.M.S.) framework [10,12,16] offers a distinct vision for integrating traditional systems like Ayurveda with modern science. Unlike previous integration attempts that often reduce traditional concepts to fit within biomedical paradigms, Co.M.S. posits that true integration requires fostering a synergistic interaction that respects and preserves the epistemological integrity of each knowledge system involved.

A core tenet of Co.M.S. is the critique of unidirectional approaches where traditional knowledge is often fragmented and analyzed solely through reductionist modern scientific methods [8,16]. While valuable for specific inquiries, this approach risks losing the holistic perspective and systemic understanding that are the hallmarks of systems like Ayurveda. Co.M.S. proposes, instead, a bidirectional exploration, seeking common ground and potential convergences while acknowledging fundamental differences in worldview and methodology.

Crucially for the present study, Co.M.S. emphasizes the value of exploring the Ayurveda-to-Modern direction [10,12]. This involves leveraging Ayurveda's comprehensive, systemic, and often qualitative framework as a lens to potentially understand and contextualize phenomena within modern medicine and pharmacology. This direction does not discount modern findings but seeks to embed them within a broader, holistic perspective, potentially revealing patterns or relationships obscured by purely mechanistic analysis.

Ultimately, Co.M.S. aims to move beyond simple validation towards a deeper understanding focused on the fundamental questions of What is working, How it is working (within both systems' perspectives), and Why it is working [12]. This article applies this Co.M.S. vision specifically to the interface between Ayurvedic principles and modern pharmacology.

### 2.2 The Ayurvedic framework: An emergent view of substance and action

Ayurveda understands the universe, including living organisms, as composed of fundamental elements and governed by universal principles [5,6]. It views reality as an interconnected network where health arises from dynamic equilibrium [17]. Central to this perspective is the concept that form is intrinsically linked to and derived from function [18,19].

At its most fundamental level, Ayurveda is primarily rooted in *Samkhya* philosophy, which provides its core metaphysical framework through the evolution of *prakriti* (primordial nature) into increasingly differentiated forms [17,20]. According to *Samkhya*, subtler vibratory states of the three guna (sattva, rajas, tamas) and their permutations lead to the manifestation of material elements [20]. Similar concepts of primordial movement or vibration (such as spanda) are also found in other Indian philosophical systems like Kashmir Shaivism [21], reflecting the rich philosophical tapestry that has influenced Ayurvedic thought over millennia. From these subtle vibratory states emerge the *Panchamahabhuta* -- *Akasha* (Space), *Vayu* (Motion), *Agni* (Transformation), *Jala* (Cohesion), and *Prithvi* (Structure) [19,22]. These are not merely physical “elements" in the Western sense, but functional states of matter with specific organizational principles that represent different patterns and potentials of energy expression.

From the inherent nature of these Mahabhuta emerge specific properties known as Guna [19,22]. The particular subset of these known as the Guruvadi Guna comprises ten pairs of opposite qualities (such as Guru/Laghu - Heavy/Light; Shita/Ushna - Cold/Hot; Snigdha/Ruksha - Unctuous/Dry), creating functional dipoles that characterize all substances and processes [22]. These paired opposites are fundamental to understanding how properties interact and balance each other in Ayurvedic thinking.

These Guna, in turn, express themselves in coherent ensembles called *Tridosha*—*Vata,* *Pitta*, and *Kapha*—the primary organizing principles governing all physiological and behavioral functions in living beings [5,7]. Rather than being separate from the *Mahabhuta* and *Guna*, the *Dosha* represent their functional manifestation in biological systems. *Vata* (primarily *Akasha* and *Vayu*), *Pitta* (primarily *Agni* and *Jala*), and *Kapha* (primarily *Jala* and *Prithvi*) each govern distinct aspects of physiology – movement, transformation, and structure, respectively.

The actual manifested action or effect, known as *Karma*, emerges from this cascade but is highly context-dependent [18]. It arises from the complex interaction between the full profile of a substance's *Guna* and the specific biological terrain of the individual, defined by:

*Prakriti*: The individual's unique, baseline constitutional balance of the *Tridosha* [6,13,14].

*Vikriti*: The current state of Doshic imbalance or deviation from *Prakriti* [6].

*Agni*: The metabolic capacity that processes substances [22].

*Ama*: Dysmetabolic residues that can interfere with proper function [23].

Importantly, this system is not merely linear but circular -- higher emergent properties can influence lower foundational elements through feedback mechanisms, creating a dynamic, constantly adjusting system [17,25]. For example, accumulated *Ama* can impact *Agni*, which affects how *Dosha* manifest, which in turn influences the expression of *Guna* within the body's tissues.

This hierarchical yet circular cascade—primordial vibration → *Mahabhuta* → *Guna* → *Dosha* → *Karma*—provides the conceptual basis for understanding how substances interact with living systems [17,18]. It aligns with the foundational Ayurvedic principle that form and structure ultimately arise from, and reflect, underlying functions and potentials within an interconnected system.

### 2.3 Proposed framework: interpreting drugs through the Ayurvedic dynamic cascade

Applying the hierarchical and circular understanding outlined above (primordial vibration → *Mahabhuta* → *Guna* → *Dosha* → *Karma*), our proposed framework interprets modern drugs by sequentially considering their properties through these Ayurvedic lenses. This interpretive process translates the structural and functional data of modern pharmacology into the qualitative, relational language of Ayurveda to generate holistic hypotheses about a drug's potential impact.

The interpretation begins at the foundational level by considering the drug's physicochemical properties and molecular structure as expressions of underlying *Panchamahabhuta* dominance (summarized in Table 1) [19,22]. For example, stable cyclic structures in a drug molecule reflect *Prithvi* predominance, while mobile aliphatic chains suggest *Vayu* qualities. This offers initial insight into the drug's fundamental nature at the level of matter organization.

**Table 1:** Pañcamahābhūta - Atomic and molecular level.

Mahabhuta	Chemical structure	Symbolic match	Modern Pharmacology examples	Ayurvedic Example & Rationale
*Prithvi*	Stable rings, cyclic structures or aromatic rings. Metal ions.	Stability solidity structure	Foundational structure	**Ashwagandha root (*Withania somnifera*)** grounding, strengthening. Contains stable steroidal lactones
*Vayu*	Mobile chains, aliphatic chains	Movement dynamism flexibility.	Molecules adaptability, dynamic interaction with biological targets, flexibility and motion. Volatile compounds. Gas-releasing molecules.	**Vacha rhizome (*Acorus calamus*)** stimulates nerve function. Contains volatile oils (asarone).
*Agni*	Electronegative atoms. Aldehydes, acids	TransformationMetabolism energy	Introduction reactivity to molecules. Raising their level of reactivity to engage energetically with the environment.	**Pippali fruit (*Piper longum*)** digestive stimulant and bioavailability enhancing properties. Contains reactive alkaloid piperine.
*Jala*	Hydrophilic groups (i.e. hydroxyls, amines, carboxyls).	Cohesion fluidity connection	Allows molecules to interact with the body's aqueous environment, facilitating dissolution, absorption, and systemic distribution.	**Licorice root (*Glycyrrhiza glabra*)** soothing, demulcent properties. Contains hydrophilic saponins (glycyrrhizin).
*Akasha*	Molecular voids, or steric gaps. Crystals.	Emptiness Void space	Capacity to fit into the target, i.e. a protein pocket or a receptor. Crucial role in drug efficacy	**Pearls (*Mukta Pishti*)** cooling, calming; processed into fine crystalline powder. Represents space/subtlety in formulation.

From these *Mahabhautik* properties naturally emerge the drug's characteristic *Guruvadi Guna* (summarized in Table 3) [19,22]. These inherent qualities—such as whether the drug has heating (*ushna*) or cooling (*shita*) properties, whether it acts rapidly (*tikshna*) or slowly (*manda*), whether it has drying (*ruksha*) or moistening (*snigdha*) effects—represent the drug's potential energetic signature or informational impact on the biological system. Understanding these paired qualities is essential for predicting how the drug might interact with similar or opposite qualities in the body.

Based on the specific combination of *Mahabhuta* and their expressed *Guna*, we can then understand the drug's likely influence on the *Tridosha* (summarized in Table 2) [5,7]. This step identifies whether the drug will primarily influence *vata's* movement functions, *Pitta's* transformative processes, *Kapha's* structural aspects, or some combination thereof.

**Table 2:** Tridosha - Cellular and systemic level.

Dosha	Symbolic match cellular level	Modern Pharmacology examples
***Vata*** (*akasha* + *Vayu*)	**Movement and transmission.** Processes like active transport across cell membranes, propagation of nerve impulses, respiration, or the systemic movement of substances	Drugs modulating neural transmission (i.e. antiepileptics), affecting respiratory rates (i.e. bronchodilators or depressants), influencing circulatory dynamics (i.e. antihypertensives). Drug distribution and metabolism. Electropositivity or positively charged atoms.
***Pitta*** (*agni* + *Jala*)	**Transformation and metabolism.** Processes like mitochondria activity, enzyme-mediated reactions, or the systemic regulation of body temperature and metabolism.	Drugs influencing metabolic rates (i.e. thyroid hormones), regulating digestive enzymes (i.e. proton pump inhibitors), modulating hormonal pathways (i.e. oral contraceptives).
***Kapha*** (*Prithvi* + *Jala*)	**Structure and cohesion.** The structural integrity of cells, the extracellular matrix, tissue formation, and the systemic aspects of protection and nourishment, such as the immune response or the maintenance of bodily fluids.	Drugs influencing fluid balances (i.e. diuretics), promoting tissue growth (i.e. growth factors) providing nourishment and strength (i.e. certain supplements). Drugs reducing edema, sluggishness, or excessive mucus production. Electronegativity or negatively charged atoms.

**Table 3:** *Guruvadi Guna* system - Directly observable properties.

*Guruvadi Guna*	Symbolic Match	Modern Pharmacology examples	Ayurvedic Example & Rationale
***Guru***	High density high complexity	Large, complex molecules with multiple functional groups, molecules with substantial weight, dense structures. Longer half-lives or prolonged effects	**Ghee (*Ghrita*)** nourishing, building qualities, increases Kapha, considered heavy to digest.
***Laghu***	Low density low complexity	Smaller molecules with simpler structures or ones that can rapidly penetrate cells and tissues. Rapidly metabolised or excreted. Volatile compounds.	**Moong dal (*Mudga*)** the lightest pulse, easy to digest, suitable during illness.
***Manda***	Extended, sustained, dull or slow action and/or release.	Drugs having a gradual onset of action, extended-release formulations, or those that work over prolonged periods. Drugs slowing down bodily functions, i.e. sedatives or tranquilisers.	***Withania somnifera* (*ashwagandha*)** adaptogen, promotes calmness and strength; building and stabilizing effects rather than rapid/sharp
***Tikshna***	Rapid, quick, fast, direct, penetrating action and/or release.	Drugs with rapid action, which can quickly cross barriers (like the blood-brain barrier) or have an immediate therapeutic impact	***Acorus calamus (Vacha*)** sharp (*tikshṇa*) quality and ability to penetrate channels, especially those related to the mind.
***Shita***	Cooling, inhibiting, contracting action	Molecules with calming, anti-inflammatory, or cooling effects, including antipyretics and anti-inflammatories.	***Emblica officinalis (Amalaki)****Rasayana*, known for its cooling potency and ability to pacify *Pitta* dosha.
***Ushna***	Warming, activating, expanding action	Molecules that stimulate metabolism or physiology, inducing warmth—e.g., thermogenics or CNS stimulants.	***Garlic (Lashuna)*** Pungent taste, heating potency, increases Pitta and digestive fire (*agni*).
***Snigdha***	Moisturising, lubricating, action	Lipophilic drugs, Hydrophobic molecules or those that enhance lubrication, moisturise tissues or used to treat dry conditions. i.e. Hyaluronic acid	**Castor Oil (*Eranda Taila*)** Thick, viscous oil with lubricating (snehana) and *Vata*-pacifying properties.
***Ruksha***	Dehydrating, hindering action	Hydrophilic molecules that are not lipid-soluble and reduce bodily fluids—e.g., diuretics or drugs with drying effects.	**Barley (*Yava*)** drying and light, good for Kapha conditions, reduces excess fluidity.
***Slakshṇa***	Soothing, harmonising, balancing, relaxing action	Agents that smooth out irregularities, such as anti-arhythmic	**Milk (*Ksheera*)** smooth, nourishing, and soothing properties, beneficial for Vata and Pitta.
***Khara***	Disrupting, abrading, wearing action	Drugs that have a scraping effect or work by dislodging or breaking down structures, like certain antitumor agents.	**Chickpea flour (Besan/Chanaka)** flour is rough-textured, drying, and often used externally for cleansing or exfoliation
***Sandra***	Compactness, high density. Compacting, packing, bulking, condensing action	Molecules with compact, densely packed structures and/or providing structure, stability. Drugs with a compact form that holds significant impact or potency, i.e. insulin	**Yogurt/Curd (*Dadhi*)** Thick, dense, semi-solid consistency, known to be channel-clogging if improperly consumed.
***Drava***	Fluidity, flexibility. Loosening, unbinding action.	Drugs that increase fluidity, such as mucolytics or expectorants. Adaptogenic agents.	**Water (*Jala*)** the essential liquid enabling fluidity, transport, and dissolution of substances
***Mridu***	Structural adaptability. Softening action.	Molecules with weak dipoles. Drugs that soften and relax tissues, i.e. emollients or topical creams.	**Aloe Vera Gel (*Kumari*)** Gelatinous pulp with soothing, cooling, and softening properties on skin and mucous membranes.
***Kathina***	Structural firmness. Hardening, stiffening, thickening action	Molecules with multiple strong dipoles. Drugs reinforcing and solidifying structures, i.e. bisphosphonates, calcium supplements	**Coral calcium (*Pravala Pishti*)** processed coral provides calcium, associated with hardness and structure.
***Sthira***	Stillness, steadiness. Braking, blocking, stabilizing action.	Molecules with stable structures, prolonged effects, or those that stabilize physiological systems, i.e. Antipsychotics or drugs for blood pressure management, such as beta-blockers	**Sida cordifolia (*Bala*)** stabilizing, strengthening and Vata-pacifying properties.
***Chala***	Mobility, instability, swiftness. Mobilising, vibratory, destabilising action	Molecules with unstable structures and rapid action that alter bodily dynamics, enhancing circulation or movement—e.g., laxatives, bronchodilators, anticoagulants.	**Terminalia chebula (*Haritaki*)** primary herb in Triphala, promoting elimination and downward movement
***Sukshma***	Subtlety fineness Pervasiveness.	Small-sized molecules acting at a subtle molecular level, capable of penetrating deep tissues or crossing barriers—e.g., nanomedicines, targeted delivery systems, monoclonal antibodies, hormone therapies.	**Asafoetida (*Hing*)** Pungent resin known for its deeply penetrating (sūkṣma) quality and ability to move Vata and clear subtle channels.
***Sthula***	Grossness, largeness. Broad, heavy, superficial or large-impact action	Drugs with pronounced, overt effects, i.e. broad-spectrum antibiotics, corticosteroids, opioid painkillers, or those that work on the superficial layers, i.e. topical treatments, skin creams, patches, wound dressings.	**Black gram (*Masha/Urad dal*)** Classically considered very heavy and bulky, difficult to digest, increases Kapha and physical bulk.
***Vishada***	Diffusiveness, non-stickiness. Clarifying, purifying action.	Water-soluble molecules, easily diffusive. Drugs with clear, precise actions and minimal side effects, i.e. nootropics or cognitive enhancers, transdermal patches	**Buttermilk (*Takra*)** light and channel-clearing fermented liquid, counteracting sliminess and stickiness.
***Picchila***	Stickyness adhesiveness, Cohesiveness.	Lipid-soluble or viscous molecules forming colloidal solutions. Drugs with gel-like consistency that adhere to surfaces and release effects slowly—i.e. mucoadhesive systems, medicated gels or ointments.	**Okra (*Bhindi*)** Mucilaginous vegetable with slimy and smooth qualities.

Finally, the framework views the drug's overall observed effects—both therapeutic efficacy and side effects—as the emergent *Karma* [18]. Crucially, this *Karma* is not determined solely by the drug's inherent properties but emerges from the complex, context-dependent interaction between the drug's entire *Guna* profile and the individual's specific physiological terrain (*Prakriti*, *Vikriti*, *Agni*, *Ama*) [15,17]. This explains why identical drugs can produce markedly different effects in different individuals.

Additionally, the circular nature of this system means that the drug's effects can create feedback loops that alter the patient's Doshic balance, *Agni* strength, or *Ama* accumulation, which then modifies how subsequent doses of the same drug are processed and expressed [17,24]. This dynamic perspective accounts for temporal changes in drug response over prolonged treatment periods.

By applying Ayurvedic principles to modern pharmacology in this way, we obtain a hierarchical, multi-tiered perspective that enables a nuanced interpretation of drug effects, accounting for the complex interplay of factors, including chemical structure, individual constitution, and environmental context. It moves beyond the reductionist view of isolated chemical constituents to a broader understanding of substances within a network of interactions [17,24].

## 3 Illustrative applications: from framework to interpretation

To demonstrate how this Co.M.S.-based framework can be practically applied, we explore its potential for interpreting modern drug classes. Rather than exhaustive analysis, these examples serve to illustrate the framework's potential for generating new hypotheses about drug action and individual variability.

### 3.1 Insights into the Ayurvedic perspective on drug side effects

From the Ayurvedic perspective proposed here, drug side effects are not viewed merely as off-target biochemical actions but as integral parts of the drug's overall emergent *Karma* within the individual's system. This holistic view suggests several factors contribute to adverse manifestations:

Firstly, the interaction might generate metabolic byproducts akin to *Ama* if the individual's *Agni* (metabolic capacity) is unable to properly process the drug, leading to systemic disturbances [23]. Secondly, the manifestation of a drug's *Karma* depends on the interplay of its entire profile of Guna with the individual's constitution (*Prakriti*) and current imbalance (*Vikriti*) [[13], [14], [15]].

For example, a *Vata*-pacifying drug, if overused, might disrupt the balance by increasing dryness (Ruksha) secondarily, manifesting adverse *Karma* particularly in a susceptible *Vata Prakriti* individual. Similarly, a drug primarily targeting *Pitta* might possess inherent Guna (e.g., Shita, Guru) that secondarily increase *Kapha*, leading to *Kapha*-related *Karma* like edema.

Understanding *Prakriti* helps anticipate how the *Karma* of a drug's *Guna* might manifest differently across the mind-body continuum in various individuals [15,26].

### 3.2 Some examples of Ayurvedic interpretation of modern drugs

#### 3.2.1 Antibiotics

Antibiotics [26], with stable rings resonating with the *Prithvi* element and reactive groups embodying Tejas, disrupt bacterial cell walls, proteins, or DNA. They primarily affect the *Pitta Dosha*, reflecting their role in transforming the internal environment to combat bacteria.

Broad-spectrum and rapid-action antibiotics may also influence Vata due to fast distribution. Bactericidal antibiotics' quick action aligns with *Tikshna Guna,* aggressively targeting bacteria, while their overall transformative effect is *Ushna Guna*. Broad-spectrum types display Ślakṣṇa quality, effectively targeting a range of bacteria.

For example, Penicillin, with its foundational beta-lactam ring (Prithvi) and reactive side chain (*Tejas/Tikshna*), effectively disrupts gram-positive bacteria's cell walls, countering their Kapha-like stability. Tetracycline targets protein synthesis with a balanced, slower (*manda*) and cooler (*shita*) action against gram-positive and gram-negative bacteria. Ciprofloxacin, a fluoroquinolone, has a penetrating action (*sukshma*) targeting gram-negative bacteria with aggressive (*tikshna*) and heating (*ushna*) qualities. Its profound effect on bacterial DNA replication aligns with Pitta Dosha and subtly with Vata, leading to significant bacterial disruption.

The manifestation of side effects might vary by *Prakriti* [15], potentially influencing both physical reactions (e.g., rashes in *Pitta* types) and subtle mental states according to Doshic influence. *Kapha* types may experience more mucus and sluggish digestion or 'heaviness' or lethargy, Vata types could face dryness and digestive irregularities, and *Pitta* types might experience inflammatory reactions.

#### 3.2.2 Antihypertensive drugs

Antihypertensive drugs [27] include diuretics, beta-blockers, ACE inhibitors, and Calcium Channel Blockers, each working through different mechanisms.

- Diuretics reduce blood pressure by helping kidneys eliminate excess salt and water; this aligns with actions on *apana* *vata* and *kapha* and inducing *laghu Guna* due to their fluid-reducing effect and *ruksha* *guna* for their dehydrating impact. For example, Furosemide and Hydrochlorothiazide have benzene rings, representing stability (*prithvi*) and sulfonyl groups for reactivity (*tejas*/*ushna*), aiding in fluid elimination. Overuse might lead to *vata* imbalance, causing dehydration or electrolyte disturbances or *vata*-related mental states like feeling 'ungrounded'.
- Beta-blockers (i.e., Atenolol) reduce heart rate and blood pressure by blocking adrenaline effects, an action closely related to *sadhaka* *pitta* and *vyana* *vata*. They stabilize heart rhythm through *sthira*, *shita* and *manda* *guna*, balancing *vata* mobility and *pitta's* influence on heart function (*sadhaka* *pitta*, linked to emotional processing). However, excessive use may cause fatigue or reduced circulation, symptoms of v*ata* aggravation.
- Angiotensin converting enzyme inhibitors (i.e., Lisinopril) by reducing angiotensin II formation, treat high blood pressure and heart failure. Their structure includes peptidyl moieties (*prithvi*) and zinc chelating groups (*tejas*), balancing *vata* by reducing vasoconstriction and *kapha* by diminishing fluid accumulation. Their overuse might disturb the *vata-kapha* balance, leading to side effects like dry cough or elevated blood urea nitrogen levels.
- Calcium Channel Blockers (i.e., Amlodipine) inhibit calcium influx, causing vasodilation. Their structure, usually featuring multiple aromatic rings (prithvi) and functional groups interacting with calcium channels, suggests stabilizing (*sthira*) and cooling (*shita*) properties. They primarily balance vata and pitta, but side effects may include edema or constipation, indicating Kapha accumulation.

In Ayurvedic terms, hypertension is often a Vata imbalance, with contributions from *pitta* and *kapha*. Each drug class must be prescribed judiciously to prevent aggravating the patient's inherent *dosha* tendencies and causing side effects.

## 4 Clinical applications

The Ayurvedic interpretation of modern drugs provides immediate practical applications for clinical practice, even as empirical validation continues [28]. These applications represent not a replacement of conventional prescribing approaches but a complementary layer that enhances therapeutic outcomes:

1. Enhanced Prescribing Strategy: Understanding a drug's *guna* profile allows clinicians to consider not just its primary mechanism of action but its overall impact on the patient's doṣic balance and to select medications that best match the patient's Prakriti (constitution) [[13], [14], [15]].

For instance:

For *Vata*-predominant individuals who tend toward dryness and variability, medications with higher Snigdha (moistening) and *Sthira* (stabilizing) qualities may be preferred when options exist.

*Pitta*-predominant patients may respond better to medications with *Shita* (cooling) properties rather than those with strong *Ushna* (heating) qualities.

*Kapha*-predominant individuals might benefit from formulations with more *laghu* (light) and *ruksha* (drying) properties.

2. Anticipatory Side Effect Management: Rather than waiting for adverse effects to manifest, practitioners can proactively implement supportive measures based on a drug's *guna* profile:

When prescribing antibiotics (with their *ushna* and *tikshna* qualities), recommending cooling herbs or foods to balance potential *pitta* aggravation.

For diuretics with strong *ruksha* (drying) qualities, suggesting appropriate hydration strategies and possibly mild *snigdha* (moistening) dietary adjustments for *vata*-predominant patients.

When using beta-blockers with Shita (cooling) and Manda (slow) qualities, monitoring for and addressing potential Kapha aggravation in susceptible individuals.

3. Synergistic Treatment Protocols: The Ayurvedic framework enables the development of comprehensive treatment protocols that address both the disease and the individual's constitutional balance [6,15]:

Combining pharmaceutical interventions with targeted dietary recommendations based on the drug's Doshic effects.

Integrating lifestyle modifications that counterbalance potential Doshic imbalances caused by necessary medications.

Timing medication administration in accordance with natural Doshic rhythms to maximize efficacy and minimize adverse effects.

4. Enhanced Patient Communication: This framework provides a language that bridges bodily experiences to systemic patterns:

Patients can better understand why they might experience certain side effects based on their constitutional tendencies.

Improved ability to articulate subjective medication experiences, leading to more effective medication adjustment.

Greater patient engagement through understanding their unique constitutional patterns and how treatments interact with these patterns.

These practical applications demonstrate how the Ayurvedic reading of modern drugs can immediately enhance clinical care, while also providing a foundation for more sophisticated research into personalized medicine approaches [15].

## 5 Discussion

This application of the Co.M.S. framework [[10], [11], [12]] proposes a significant enhancement to our understanding of modern drugs by incorporating the comprehensive epistemological perspective derived from Ayurvedic principles. By mapping drug properties to Guna and interpreting effects as emergent Karma within the individual's *Prakriti*/*Vikriti* context, we gain a multi-layered understanding that supplements and amplifies—rather than replaces—conventional mechanism of action analysis.

The synthesis of Ayurveda with modern pharmacology represents a transformative integration of two complementary approaches to understanding therapeutic agents [17]. This integrated perspective does not diminish the value of precise molecular understanding but rather contextualizes it within a broader systems view that better explains individual variability in drug response [24].

Modern pharmacology offers granular understanding with its molecular precision, but Ayurveda's broader view captures a drug's ripple effects through the body's ecosystem [17,24]. Mechanism of action explains target interaction; the Ayurvedic interpretation explores how this interaction manifests as overall physiological action (*karma*) within the individual's unique, complex system, adding crucial context for therapeutic outcomes.

This approach advocates for a therapeutic approach that considers not just the pharmacological properties of drugs but also the intricate interplay between these properties, the individual's constitution (*prakriti*), and the environmental milieu [15,17]. The Co.M.S. approach provides practical tools for understanding why different individuals (*prakriti*) experience the *karma* of a specific drug differently, offering a path toward more nuanced personalization.

While empirical validation will strengthen this framework [28], its value is not solely dependent on such validation. The conceptual coherence of mapping modern drug properties to Ayurvedic categories already provides valuable insights that can inform clinical decision-making and enhance patient care. The framework does not require abandoning conventional pharmacological knowledge but rather enriches it by situating it within a more comprehensive understanding of human physiology and individual variation.

### 5.1 Integrating the mind-body continuum: form as manifested meaning

A fundamental tenet of Ayurveda is the inseparable nature of mind and body [6,26]. In this view, the physical body can be understood as the tangible form (*rupa*) that emerges from and symbolically represents the underlying semantic potential and processes of the mind (*nama*), echoing the principle that form follows function, or more deeply, meaning [17].

The Co.M.S. framework allows us to explore these psycho-physiological links [26]. When interpreting a drug through its influence on *dosha* and *guna*, we assess its potential impact not just on physical functions, but on the entire psycho-physiological complex, viewing the physical effects as potential downstream manifestations of changes initiated at a more subtle level.

For instance, a drug interpreted as having a potent *pitta*-pacifying *karma* might address physical inflammation while simultaneously calming associated mental states like irritability or intensity. This integrated view suggests that modern drugs, though designed for specific molecular targets, inevitably interact with the holistic mind-body system.

### 5.2 Limitations and boundaries of the framework

As a discusion kernel article introducing a novel conceptual framework, we acknowledge several important limitations:

1. Interpretive Nature: The mappings between modern pharmacological properties and Ayurvedic categories are interpretive rather than deterministic [9]. These connections represent hypothesized relationships that require empirical validation.
2. Complexity of Translation: The translation between epistemological systems inevitably involves some degree of conceptual stretching [9]. Not all modern pharmacological concepts find perfect analogs in Ayurvedic thinking, and vice versa.
3. Validation Requirements: While our framework offers explanatory potential, its predictive power remains to be established through rigorous clinical studies that correlate Prakriti assessments with measured drug responses [28].
4. Scope Boundaries: This approach is likely most applicable to understanding variability in drug response and side effect profiles rather than replacing molecular mechanism studies or pharmacokinetic analyses.

It is important to acknowledge that while this framework offers a coherent conceptual approach to understanding drug actions through Ayurvedic principles, systematic empirical validation remains a critical next step. The correspondences proposed between modern pharmacological properties and Ayurvedic categories represent theoretical constructs that, though internally consistent and grounded in both traditional wisdom and contemporary understanding, require rigorous experimental verification. Future studies should specifically investigate whether *prakriti*-based prescribing approaches result in measurably improved clinical outcomes, reduced side effect profiles, or enhanced patient satisfaction compared to conventional approaches. This validation gap does not diminish the value of the conceptual framework itself, which serves as an essential foundation for generating testable hypotheses and guiding research design. Rather, it highlights the opportunity for a productive research agenda that could strengthen the bridge between traditional wisdom and evidence-based practice. The framework's primary contribution at this stage is providing a structured, coherent approach to reimagining pharmacological actions within an Ayurvedic epistemology, setting the stage for empirical work that may substantiate, refine, or reconfigure aspects of this initial theoretical model.

Despite these limitations, we believe the framework offers significant value as a complementary perspective that can generate novel hypotheses and potentially enhance clinical decision-making even before complete validation.

## 6 Conclusion

Applying the Co.M.S. framework [10,12] to interpret modern pharmacology through Ayurvedic principles offers a novel, integrative perspective that enhances our ability to prescribe medications effectively and personalize treatment approaches. By viewing drugs through the lens of Panchamahabhuta, Tridosha, and Guna, and understanding their effects as emergent Karma within the individual's constitutional context (Prakriti/Vikriti), this approach provides practical tools to explore drug actions holistically.

Our approach—that Ayurvedic epistemology provides a more inclusive framework for understanding modern drug actions—offers significant advantages for clinical practice and explains the persistent challenge of inter-individual variability in drug response [1,2,15]. This perspective does not replace modern pharmacological understanding but supports and amplifies it by adding deeper layers of interpretation.

The practical applications of this approach are numerous: optimizing prescription practices based on constitutional considerations, reducing side effects through anticipatory management, creating synergistic treatment protocols, and enhancing patient communication and engagement. While empirical validation will further strengthen this framework, it already offers valuable insights that can immediately benefit patient care.

While recognizing that empirical validation through clinical studies represents a necessary future direction, this kernel article establishes the conceptual groundwork that makes such validation studies both possible and meaningful by providing clear, testable propositions about how Ayurvedic principles might enhance our understanding of modern drug actions. This article invites the scientific and medical communities to explore this framework's potential through both practical clinical application and systematic research, potentially leading to a more integrated understanding of how medicines work within the complex human system and improving healthcare outcomes for diverse patient populations.

## Author contrubutions

All the authors were involved in conceptualization, study methods and related investigations. AM supervised the work and wrote the first draft. All authors reviewed and approved the draft.

## Declaration of generative AI in scientific writing

AI has been used solely for refining the language and improving clarity, as English is not my first language. The ideas, structure, and content of the article remain entirely my own.

## Funding sources

None.

## Conflict of interest

The authors declare that they have no known competing financial interests or personal relationships that could have appeared to influence the work reported in this paper.
